# Surgeon preferences and practice patterns in rectopexy: Results of an international survey

**DOI:** 10.1111/codi.70355

**Published:** 2026-01-04

**Authors:** Eleftherios Gialamas, Isabelle Uhe, Pierre‐Alain Tokoto, Emilie Liot, Frédéric Ris, Steven D. Wexner, Jeremy Meyer, Guillaume Meurette, the International Rectopexy Collaborative Group, Abdulahad Al‐Ameri, Adam Mylonakis, Ahmed Abdelsamad, Aakansha Giri Goswami, Al Marazgh Mohammad, Alberto Aiolfi, Alessia Fassari, Angelo Alessandro Marra, Alessandro Garcea, Alexandra Menni, Alexandra Winter, Alexandros Chamzin, Alexandros Kozadinos, Alice Frontali, Amine Gouader, Amine Souadka, Amanda Pereira Lima, Andrea Balla, Andrea Peloso, Andrea Pierre Luzzi, Andreas Panagakis, Andrei Chitul, Andrei Popa, Angelo Stuto, Angeliki Vouchara, Antonio Castaldi, Antonio Luberto, Arcangelo Picciariello, Argyrios Ioannidis, Augustinas Bausys, Audrius Dulskas, Baljit Singh, Benjamin Fernandez, Boris Schiltz, Bruno Perotti, Bruno Roche, Brunella M. Pirozzi, Carlos Augusto Gomes, Carmen Gorgan, Céline Duvoisin Cordoba, Charles Sabbagh, Christina A. Fleming, Christos Barkolias, Christos Chouliaras, Christoph Werner Strey, Claudio Soravia, Cosimo R. Scarpa, Dan‐Eduard Giuvara, Danilo Vinci, David Alessio Merlini, David D.E. Zimmerman, Debby Keller, Deiro Giacomo, Dieter Hahnloser, Diletta Corallino, Dimitris Korkolis, Dimitrios Linardoutsos, Dimitrios Moris, Dimitrios Schizas, Eleandros Kyros, Elisa Reitano, Elissavet Anestiadou, Emanuela Silva Alvarenga, Eric G. Weiss, Fabio Carbone, Federica Di Marco, Filippo Carannante, Flaviu Ionut Faur, Floryn Cherbanyk, Francesco Ferrara, Francesco Pata, Gaetano Florio, Gabriele Naldini, Gabriele Pozzo, Gabriella Teresa Capolupo, Giacomo Ambrogi, Giacomo Calini, Gianluca Pellino, Gianpiero Gravante, Giovanna Dasilva, Giovanni Cestaro, Giovanni Tebala, Giovanni Tomasicchio, Giuseppe Brisinda, Giuseppe Candilio, Giuseppe Giuliani, Goran Barisic, Goytom Knfe, George Stavrou, Georgios Korovesis, Georgios Peros, Georgios Tzikos, Gustavo Nari, Harish Neelamraju Lakshmi, Hemendra Kumar Mangal, Hugh M. Paterson, Ibrahim Burak Bahçecioğlu, Ibrahim Ethem Gecim, İlgar Ismayilov, Ioannis Katsaros, Jasper Stijns, Jelenko Jelenkovic, Jin Jiun Mah, Justin Davies, Kashish Malhotra, Klaus Peitgen, Konstantinos Tsimogiannis, Konstantinos Zarras, Lars Thomas Seeberg, Leandro Siragusa, Lorenzo Epis, Lucio Taglietti, Lysandros Karydakis, Maria Chiara Ranucci, Maria Sotiropoulou, Mark Potter, Marko Miladinov, Martin Bertrand, Matteo Santoliquido, Mhairi Collie, Michel Adamina, Michaela Ramser, Michaël Racine, Michail Vailas, Mohamed Ali Chaouch, Mohd. Azharuddin Attar, Mostafa Shalaby, Muhammad Rafaih Iqbal, Muhammad Umar Younis, Mustafa Yener Uzunoglu, Niels Komen, Nicola Colucci, Nicolas C. Buchs, Nir Horesh, Noam Shussman, Nora Abbesorabi, Nuri Okkabaz, Omer Yalkin, Orestis Ioannidis, Paolo Ossola, Paolo Panaccio, Patricia Tejedor, Pedro Botelho, Pietro Fransvea, Pim B. Olthof, Pravin Meenashi Sundaram, Priscila Tanuri, Prokopis Christodoulou, Raffaele Galli, Raja Basit Khan, Rémy Kohler, Renan Colombari, Rogier Crolla, Sarah Vogler, Saulius Mikalauskas, Sameh Emile, Sergio Agradi, Sergio Larach, Sevket Baris Morkavuk, Simone Manfredelli, Spyridon Davakis, Stefano Gussago, Stefano Olmi, Stephan Bischofberger, Suman Baral, Syed Muhammad Ali, Tahar Fillali, Valentin Calu, Valentina Miacci, Venkatesh Munikrishnan, Vittoria Bellato, Vusal Aliyev, William Perry, Xenofon Papazarkadas, Yasuko Maeda

**Affiliations:** ^1^ Division of Abdominal Surgery Geneva University Hospitals Geneva Switzerland; ^2^ Medical School University of Geneva Geneva Switzerland; ^3^ Department of Surgery MedStar Georgetown University Hospital Washington DC USA

**Keywords:** rectal prolapse, rectopexy, surgeon's preference, survey

## Abstract

**Aim:**

Rectopexy is the preferred abdominal intervention for rectal prolapse. Despite similar procedural steps – rectal mobilisation, prolapse reduction, and fixation – techniques vary widely, and onsensus on the optimal approach is lacking. This study aimed to assess global surgeon preferences and practices in rectopexy.

**Methods:**

An international 28‐item online survey was distributed between November 2023 and March 2024 through professional networks and social media. Questions addressed surgeon demographics, perioperative strategies, and technical approaches to rectopexy. Responses were analysed descriptively and stratified by region and specialty.

**Results:**

A total of 226 surgeons from 36 countries across four continents completed the survey. Most respondents (79.6%) administered preoperative intravenous antibiotics, and 80.5% used some form of mechanical bowel preparation. Minimally invasive approaches predominated (81%), with laparoscopy being most common. Posterior dissection was preferred by 61.5%, while 38.5% favoured ventral (anterior) dissection. Two‐thirds (68.1%) routinely used mesh, predominantly synthetic. Only 15% performed rectopexy as a day‐case procedure. Regional and specialty‐related variations were evident in approach, mesh type, and perioperative protocols.

**Conclusion:**

This international survey reveals marked variability in rectopexy practice worldwide. Despite common principles, surgeon preference and regional factors strongly influence decision‐making. The findings emphasise the need for updated international guidelines to harmonise technique selection and perioperative management in rectal prolapse surgery.

**What does this paper add to the literature?**

This international survey identifies substantial global variation in rectopexy techniques and perioperative management for rectal prolapse. It highlights that, despite consensus efforts, surgical practice remains driven largely by individual and regional preferences, underscoring the urgent need for updated, evidence‐based guidelines to standardise rectopexy practice worldwide.

## INTRODUCTION

Abdominal rectopexy is the preferred operative treatment for full‐thickness rectal prolapse in medically fit patients [1]. Minimally invasive approaches have largely displaced open surgery, offering reduced morbidity and faster recovery without clear detriment to recurrence or functional outcomes [2, 3]. Yet, substantial variation persists in technical conduct – including ventral versus posterior rectopexy, the extent of rectal mobilisation, mesh use and type, and methods of fixation – as well as in perioperative protocols (bowel preparation, antibiotic prophylaxis, lateral ligament preservation, drains, and discharge pathways) [4, 5, 6].

In areas where high‐quality comparative data are limited or conflicting, such heterogeneity likely reflects appropriate clinical equipoise rather than a deficiency in care. Recent consensus efforts – first through a European modified Delphi (2021), and more recently, an international expert panel (2025) – have begun to articulate best practice principles while acknowledging legitimate areas of uncertainty and the need for individualised decision‐making [5, 7]. In parallel, technical refinements (e.g., minimally invasive ventral rectopexy, selective use of biological prostheses, or strategies that avoid rectal bites) are being explored with the aim of maintaining efficacy while minimising mesh‐related risk [3, 8].

Against this backdrop, we conducted an international survey to characterise current rectopexy practice across surgeon backgrounds and regions, spanning preoperative preparation, intraoperative technique, and postoperative pathways. By mapping real‐world preferences and patterns of care, this study provides foundational data to inform future trials, guide consensus harmonisation, and identify priority questions where stronger evidence is most needed.

## METHODS

### Study design and participants

We conducted an international, cross‐sectional, anonymous online survey of surgeons who perform or manage rectopexy for full‐thickness rectal prolapse. Eligible respondents were practicing general surgeons and colorectal surgeons involved in the operative and perioperative care of rectal prolapse. Participation was voluntary, with no incentives. Reporting follows CHERRIES (Checklist for Reporting Results of Internet E‐Surveys) [9].

### Questionnaire development and pretesting

A 28‐item questionnaire was developed through a structured process. First, we conducted a focused review of the literature – including prior surveys, expert consensus statements, and international guidelines on rectal prolapse. Draft items were generated to capture: demographics, preoperative preparation (e.g., bowel preparation, antibiotics), intraoperative technique (operative approach, dissection plane, mesh use/type, fixation strategy, peritonealisation, drains, local infiltration, catheterisation), and postoperative pathways (laxatives, enema, ambulatory vs. admission, physiotherapy/biofeedback).

The initial item pool underwent expert review by four colorectal consultants in Switzerland, each with substantial experience in rectal prolapse surgery. This iterative feedback process ensured face and content validity, refined wording, and confirmed the relevance and completeness of items. The questionnaire was then pretested informally by these consultants to assess clarity and flow. Minor adjustments were made based on their comments. No formal psychometric testing (e.g., test–retest reliability) was undertaken, consistent with the descriptive, practice‐mapping aim of the survey.

### Survey administration

The survey was hosted on a secure online platform and disseminated through surgical societies, colorectal interest groups, professional mailing lists, and social media (Twitter/X, LinkedIn) between November 2023 and March 2024. Platform settings limited multiple submissions per device. To preserve anonymity, IP addresses were not stored. Because the survey link was shared across open professional networks and the denominator of invitations was unknown, a response rate could not be calculated (convenience sampling). To mitigate response bias, the survey was anonymous, IP addresses were not collected, and the distribution strategy emphasised broad dissemination across multiple professional networks.

### Measures and variable handling

All variables are reported using the text labels shown to respondents (e.g., ‘IV / Oral / No’ for antibiotics; ‘Open/Laparoscopic/Robotic’ for approach). Region was analysed at the continent level (Europe, Asia, Africa, North America, South America). Where an item allowed multiple selections, analyses reflected the predominant (most common) practice as reported. Missing data were handled with pairwise deletion at the item level; no imputation was performed.

### Outcomes

The primary objective was to describe contemporary rectopexy practice across the perioperative pathway. Exploratory secondary analyses compared practices by specialty (general vs. colorectal), region, and experience. The experience split (<50 vs. ≥50 rectopexies) was defined post hoc for exploratory purposes (informed by contemporary consensus on learning curves) and is reported as such [7].

### Statistical analysis

Categorical variables are presented as *n* (%); continuous variables as median [IQR]. Univariate comparisons used Pearson's χ^2^ or Fisher's exact test, as appropriate. Given the exploratory nature, no adjustment for multiple testing was applied; two‐sided *p* < 0.05 was considered significant. Analyses were conducted in R (R Foundation for Statistical Computing, Vienna, Austria).

### Ethical considerations

The survey was anonymous and collected no patient‐level data. Completion constituted implied consent. Per institutional policy, this study met criteria for waiver of formal ethics review for anonymous professional surveys.

## RESULTS

### Participants

**FIGURE 1:**
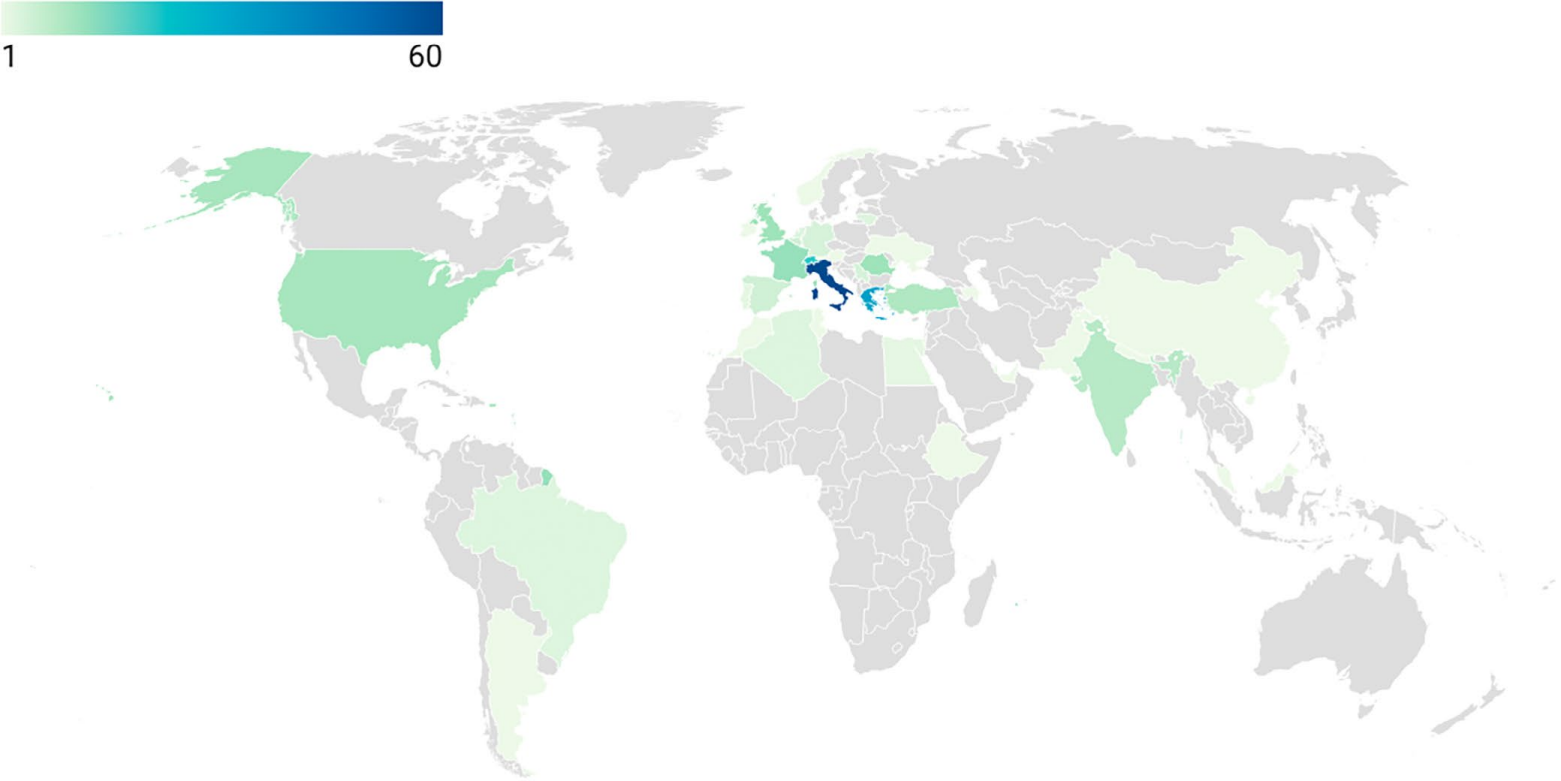
Global distribution of respondents by country (Choropleth map illustrating the number of participating surgeons per country. Darker shading corresponds to higher respondent numbers (range 1–60), while countries in grey had no participants).

A total of 226 surgeons from 36 different countries completed the survey (median age 47 years [IQR 38–56]); 138 (61.1%) were male and 88 (38.9%) were female (Table 1). Most respondents practiced in Europe (158, 70.0%), followed by North America (25, 11.1%), Asia (21, 9.3%), South America (12, 5.3%), and Africa (10, 4.4%). The international distribution of surgeons is depicted in Figure 1 (global distribution) and Supplementary Figure S1 (distribution by country). The majority were consultants (177, 78.3%) and 49 (21.7%) were residents. Two‐thirds were general surgeons (150, 66.4%) and one‐third were colorectal surgeons (76, 33.6%). Most worked in academic/university hospitals (146, 64.4%), with 61 (26.9%) in public non‐university and 20 (8.8%) in private hospitals. Years in specialised practice were <5 (23, 10.2%), 5–10 (52, 23.0%), 11–20 (76, 33.6%), and >20 (75, 33.2%). Reported lifetime rectopexy volume was <20 (49, 21.7%), 20–50 (61, 27.0%), 51–100 (42, 18.6%), and >100 (74, 32.7%).

**TABLE 1:** Demographic characteristics of respondents.

Variable	Category	*n* (%)
Age (years), median [IQR]		38 [34–45]
Gender	Male	186 (82.3%)
	Female	40 (17.7%)
Origin	Europe	180 (79.6%)
	Asia	24 (10.6%)
	Africa	8 (3.5%)
	North America	10 (4.4%)
	South America	4 (1.8%)
Position	Resident	49 (21.7%)
	Consultant	177 (78.3%)
Specialty	General surgeon	150 (66.4%)
	Colorectal surgeon	76 (33.6%)
Type of hospital	Public academic	153 (67.7%)
	Public non‐academic	37 (16.4%)
	Private	36 (15.9%)
How many years of specialised practice do you have?	<10 years	148 (65.5%)
	10–20 years	45 (19.9%)
	20–30 years	33 (14.6%)
	>30 years	0 (0.0%)
How many rectopexies have you performed in your career	<50	163 (72.1%)
	50–100	33 (14.6%)
	100–200	18 (8.0%)
	>200	12 (5.3%)

*Note*: Percentages exclude missing responses per item.

### Pre‐ and intraoperative practice

Antibiotic prophylaxis was routine: IV in 180 (79.6%), oral in 30 (13.3%), and none in 16 (7.1%) (Table 2). Bowel preparation varied: oral regimen in 99 (43.8%), enema in 83 (36.7%), and no preparation in 44 (19.5%). The preferred operative approach was predominantly minimally invasive – laparoscopic in 140 (61.9%) and robotic in 43 (19.0%) – with open surgery reported by 43 (19.0%). Posterior dissection was most common (139, 61.5%), with ventral (anterior) dissection in 87 (38.5%). Systematic mesh use was reported by 154 (68.1%), while 72 (31.9%) do not routinely use mesh. Among those declaring a predominant mesh choice, synthetic was most frequent (153, 67.7%) and biological less common (32, 14.2%). Peritoneal flap closure was standard (196, 86.7%). Most surgeons did not leave a drain (159, 70.4%), used local wound infiltration (128, 56.6%), and inserted a urinary catheter (192, 85.0%).

**TABLE 2:** Responses of pre‐ and intraoperative aspects.

Variable	Category	*n* (%)
Do you administer preoperative antibiotics?	IV	180 (79.6%)
	Oral	30 (13.3%)
	No	16 (7.1%)
Do you administer mechanical bowel preparation before rectopexy?	Enema	83 (36.7%)
	Oral	99 (43.8%)
	No	44 (19.5%)
What is your preferred method of performing rectopexy (>50% of cases over the last year)?	Open	43 (19.0%)
	Laparoscopic	140 (61.9%)
	Robotic	43 (19.0%)
Which is your preferred type of rectal dissection?	Anterior	87 (38.5%)
	Posterior	139 (61.5%)
	Lateral	0 (0.0%)
Do you use systematically a mesh or not?	Yes	154 (68.1%)
	No	72 (31.9%)
If you use a mesh, which type of mesh do you use in most cases?	Synthetic	153 (67.7%)
	Biologic	32 (14.2%)
	No	41 (18.1%)
If you use a mesh, in which position do you place it in most cases (one or more possible answers)?	Anterior	111 (68.1%)
	Posterior	36 (22.1%)
	Lateral	16 (9.8%)
If you use a mesh, how do you fix it in most cases distally?	Absorbable suture	91 (58.7%)
	Non‐absorbable suture	50 (32.3%)
	Tack	11 (7.1%)
	Glue	3 (1.9%)
If you use a mesh, how do you fix it in most cases on the promontory?	Absorbable suture	25 (13.5%)
	Non‐absorbable suture	95 (51.4%)
	Tack	61 (33.0%)
	Glue	4 (2.2%)
Do you close the peritoneal flap in most cases?	Yes	196 (86.7%)
	No	30 (13.3%)
Do you leave a drain in the end of the operation?	Yes	67 (29.6%)
	No	159 (70.4%)
Do you perform local infiltration of the surgical wound(s)?	Yes	128 (56.6%)
	No	98 (43.4%)
Do you systematically insert a urinary catheter?	Yes	192 (85.0%)
	No	34 (15.0%)

*Note*: Percentages exclude missing responses per item.

### Postoperative pathways

Routine laxatives were prescribed by 56 (24.8%); 101 (44.7%) do not prescribe them systematically and 69 (30.5%) use them selectively (Table 3). A postoperative enema was reported by 201 (88.9%). Most cases were managed with inpatient admission (192, 85.0%); ambulatory pathways were used in 34 (15.0%). Postoperative perineal physiotherapy/biofeedback was reported by 143 (63.3%).

**TABLE 3:** Responses of postoperative care.

Variable	Category	*n* (%)
Do you prescribe systematically laxatives?	Yes	101 (44.7%)
	No	56 (24.8%)
	Selected	69 (30.5%)
Do you perform systematically a enema in the postoperative course?	Yes	25 (11.1%)
	No	201 (88.9%)
The majority of cases are ambulatory or admission basis?	Hospitalisation	192 (85.0%)
	Ambulatory	34 (15.0%)
Do all patients receive postoperative perineal physiotherapy/biofeedback?	Yes	83 (36.7%)
	No	143 (63.3%)

*Note*: Percentages exclude missing responses per item.

### Between‐group comparisons

Univariate comparisons were performed across region of practice (Europe, Asia, Africa, North America, South America) and specialty (general vs. colorectal).

#### Region (continent of practice)

Bowel preparation modality differed by region (*p* < 0.05) (Table S1). Robotic use was highest in Asia, whereas laparoscopy predominated elsewhere (*p* < 0.001). Peritoneal flap closure and drain usage also varied by region (both *p* < 0.05), and ambulatory pathways were more common in North/South America than in Europe (*p* < 0.05). Interpretation should consider smaller sample sizes outside Europe.

#### Specialty (general vs. colorectal)

Colorectal surgeons reported significantly less open and greater robotic use than general surgeons (*p* < 0.05) (Table S2). They also left drains less often and used local wound infiltration more frequently (both *p* < 0.05). Mesh policy, mesh type, dissection plane, peritoneal closure, and catheterisation did not differ significantly.

#### Experience (years of specialised practice)

Differences across experience levels were observed for several perioperative choices (Table S3). Surgeons with ≥20 years of specialised practice reported less open surgery and greater adoption of robotic and laparoscopic approaches (*p* < 0.01). Systematic mesh use and preference for biological materials were more frequent among the most experienced surgeons (both *p* < 0.05). Experienced surgeons also closed the peritoneal flap more consistently and used drains less often (both *p* < 0.05).

#### Case volume (rectopexies performed)

Case volume also influenced several intra‐ and postoperative practices (Table S4). High‐volume surgeons (>200 cases) performed almost exclusively minimally invasive rectopexies, used drains less frequently, and applied local wound infiltration more often (all *p* < 0.05). No significant association was found between case volume and the use of laxatives, enemas, or postoperative physiotherapy.

## DISCUSSION AND CONCLUSIONS

This international survey demonstrates substantial practice variation in abdominal rectopexy – across operative approach, dissection plane, mesh strategy, and perioperative care – despite the predominance of minimally invasive surgery worldwide. In our cohort, most respondents favoured laparoscopy, and to a lesser extent, robotics, with nearly one in five still reporting open rectopexy. These patterns mirror the literature showing faster recovery with minimally invasive approaches compared with open repair, without clear evidence of worse recurrence or morbidity [1, 2, 10]. They also align with core recommendations of the American Society of Colon and Rectal Surgeons (ASCRS) Clinical Practice Guidelines for Rectal Prolapse [11], which endorse minimally invasive abdominal repair for medically fit patients. Consistent with our findings, a recent multiregion survey of 249 colorectal surgeons also documented wide global variation in rectal prolapse surgery, including preference for minimally invasive abdominal repair in healthy patients and regional differences in approach selection [12].

The distribution between posterior and ventral (anterior) dissection in our data reflects ongoing clinical equipoise. Contemporary guidance – including the 2014 Ventral Rectopexy Consensus [6], the ASCRS Clinical Practice Guidelines [11], the 2021 European modified Delphi [5], as well as the 2025 International Expert Panel Consensus [7] – converges on the nerve‐sparing rationale for ventral rectopexy, particularly in patients with baseline constipation or anterior compartment symptoms. At the same time, all these frameworks emphasise that dissection plane selection should be individualised according to patient phenotype and surgeon expertise. The heterogeneity in our sample likely reflects these nuanced recommendations. From an outcome perspective, posterior mobilisation, especially when lateral ligaments are divided, has been associated with a greater risk of postoperative constipation, whereas ventral dissection may better preserve autonomic integrity. Conjecturally, differences in training exposure, institutional culture, and surgeon prioritisation of functional outcomes may each contribute to the patterns observed across specialties and regions.

Practice variation was also evident in mesh strategy. Two‐thirds of mesh users reported a preference for synthetic material, with biological alternatives used less frequently. This aligns with historical practice patterns and a systematic review reporting durable outcomes with either material, set against a low but relevant risk of mesh‐related complications [7, 13, 14]. A focused review of erosion specifically suggested lower erosion with biological mesh than with synthetic mesh, though comparative data remain largely observational [13]. The 2025 International Expert Panel Consensus also advises careful attention to fixation – particularly the use of absorbable sutures and avoidance of permanent braided materials – to mitigate erosion risk [7]. Our findings sit within a broader global context in which mesh choice also varies by region (e.g., synthetic favoured in the Americas vs. biological in Australasia), reinforcing that heterogeneity is not solely European [10, 12, 15]. The variability we observed in mesh type and fixation method may have tangible clinical implications, particularly concerning erosion risk, pain syndromes, and long‐term mesh tolerance. Such differences likely reflect a combination of surgeon familiarity, resource availability, local procurement policies, and medico‐legal environment rather than differences in evidence interpretation alone.

Perioperative pathways were heterogeneous in our sample: bowel preparation modality differed by region, peritonealisation was routine, and policies on drains, local infiltration, and catheters varied. Such variation is also described in prior consensus summaries [5, 6, 7]. Some of this variation may influence early postoperative outcomes – including ileus, urinary retention, or pain control. For example, general surgeons reported greater drain use than colorectal surgeons, a difference that may reflect divergent training paradigms and interpretations of operative risk rather than evidence‐based guidance. Similarly, the limited standardisation in laxative and enema use after rectopexy could contribute to variability in postoperative bowel function. Importantly, these country‐ and specialty‐level differences suggest that system‐level factors and subspecialty culture may drive practice as much as the published evidence.

Experience and procedural volume were both associated with more standardised and minimally invasive practices, reflecting a learning curve effect and convergence towards established expert preferences. These findings align with international consensus recommendations, emphasising the role of experience in improving dissection quality, mesh handling, and functional outcomes [7]. Cumulatively, these trends suggest that higher case exposure may progressively drive harmonisation of technique and adherence to evidence‐based perioperative routines in rectopexy.

Strengths of this study include its international scope, granular capture of technique across the full perioperative pathway, and the participation of surgeons with varying backgrounds. However, several limitations should be acknowledged. Our respondent cohort was predominantly European, which may limit generalisability and underrepresent practice patterns in regions with broader access to robotics or different institutional infrastructures. In line with this, our U.S. co‐authors noted that the results may not fully reflect practice in high‐volume North American centres, particularly regarding institutional resources and adoption of robotic platforms. Practice in the USA is highly heterogeneous including university hospitals, clinics, and both private for‐profit and non‐profit hospitals, across a wide range of diverse socio‐economic and population density areas. Nonetheless, the parallel signals observed in non‐European cohorts – regarding approach, mesh selection, and perineal vs. abdominal preferences – support the external validity of our main observations [12]. The survey relied on self‐reported practices, which introduces recall and desirability bias, and our analyses were univariate, limiting causal inference. Future work should broaden participation beyond Europe, adopt core descriptor sets, and incorporate multivariable and longitudinal analyses to clarify how specialty and region influence technique selection and outcomes.

Overall, our findings – considered alongside contemporary consensus and reviews – support the view that current variability often reflects legitimate clinical equipoise rather than a deficiency in care. Crucially, what appears to matter most is correct indication (patient selection) and structured follow‐up rather than the micro‐details of technique per se. Priorities for research include comparative evaluation of dissection plane by symptom profile, mesh type and fixation (including suture choice), and standardised perioperative bundles (e.g., ERAS elements, catheter/drain policies) that might enable wider, safe use of ambulatory pathways.

## AUTHOR CONTRIBUTIONS

**Eleftherios Gialamas:** Conceptualization; investigation; writing – original draft; methodology; validation; visualization; data curation. **Isabelle Uhe:** Methodology; writing – review and editing; validation. **Pierre‐Alain Tokoto:** Writing – review and editing; validation. **Emilie Liot:** Writing – review and editing; validation. **Frédéric Ris:** Writing – review and editing; validation. **Steven D. Wexner:** Validation; writing – review and editing; supervision. **Jeremy Meyer:** Writing – review and editing; validation; supervision. **Guillaume Meurette:** Writing – review and editing; validation; supervision.

## FUNDING INFORMATION

The authors have no funding source to declare.

## CONFLICT OF INTEREST STATEMENT

The authors have no conflict of interest to disclose.

## ETHICS STATEMENT

Not applicable.

## Supporting information


Appendix S1.

Figure S1.

Table S1.

## Data Availability

The data that support the findings of this study are available from the corresponding author upon reasonable request.

## APPENDIX A

### International Rectopexy Collaborative Group

Abdulahad Al‐Ameri, Adam Mylonakis, Ahmed Abdelsamad, Aakansha Giri Goswami, Al Marazgh Mohammad, Alberto Aiolfi, Alessia Fassari, Angelo Alessandro Marra, Alessandro Garcea, Alexandra Menni, Alexandra Winter, Alexandros Chamzin, Alexandros Kozadinos, Alice Frontali, Amine Gouader, Amine Souadka, Amanda Pereira Lima, Andrea Balla, Andrea Peloso, Andrea Pierre Luzzi, Andreas Panagakis, Andrei Chitul, Andrei Popa, Angelo Stuto, Angeliki Vouchara, Antonio Castaldi, Antonio Luberto, Arcangelo Picciariello, Argyrios Ioannidis, Augustinas Bausys, Audrius Dulskas, Baljit Singh, Benjamin Fernandez, Boris Schiltz, Bruno Perotti, Bruno Roche, Brunella M. Pirozzi, Carlos Augusto Gomes, Carmen Gorgan, Céline Duvoisin Cordoba, Charles Sabbagh, Christina A. Fleming, Christos Barkolias, Christos Chouliaras, Christoph Werner Strey, Claudio Soravia, Cosimo R. Scarpa, Dan‐Eduard Giuvara, Danilo Vinci, David Alessio Merlini, David D.E. Zimmerman, Debby Keller, Deiro Giacomo, Dieter Hahnloser, Diletta Corallino, Dimitris Korkolis, Dimitrios Linardoutsos, Dimitrios Moris, Dimitrios Schizas, Eleandros Kyros, Elisa Reitano, Elissavet Anestiadou, Emanuela Silva Alvarenga, Eric G. Weiss, Fabio Carbone, Federica Di Marco, Filippo Carannante, Flaviu Ionut Faur, Floryn Cherbanyk, Francesco Ferrara, Francesco Pata, Gaetano Florio, Gabriele Naldini, Gabriele Pozzo, Gabriella Teresa Capolupo, Giacomo Ambrogi, Giacomo Calini, Gianluca Pellino, Gianpiero Gravante, Giovanna Dasilva, Giovanni Cestaro, Giovanni Tebala, Giovanni Tomasicchio, Giuseppe Brisinda, Giuseppe Candilio, Giuseppe Giuliani, Goran Barisic, Goytom Knfe, George Stavrou, Georgios Korovesis, Georgios Peros, Georgios Tzikos, Gustavo Nari, Harish Neelamraju Lakshmi, Hemendra Kumar Mangal, Hugh M. Paterson, Ibrahim Burak Bahçecioğlu, Ibrahim Ethem Gecim, İlgar Ismayilov, Ioannis Katsaros, Jasper Stijns, Jelenko Jelenkovic, Jin Jiun Mah, Justin Davies, Kashish Malhotra, Klaus Peitgen, Konstantinos Tsimogiannis, Konstantinos Zarras, Lars Thomas Seeberg, Leandro Siragusa, Lorenzo Epis, Lucio Taglietti, Lysandros Karydakis, Maria Chiara Ranucci, Maria Sotiropoulou, Mark Potter, Marko Miladinov, Martin Bertrand, Matteo Santoliquido, Mhairi Collie, Michel Adamina, Michaela Ramser, Michaël Racine, Michail Vailas, Mohamed Ali Chaouch, Mohd. Azharuddin Attar, Mostafa Shalaby, Muhammad Rafaih Iqbal, Muhammad Umar Younis, Mustafa Yener Uzunoglu, Niels Komen, Nicola Colucci, Nicolas C. Buchs, Nir Horesh, Noam Shussman, Nora Abbesorabi, Nuri Okkabaz, Omer Yalkin, Orestis Ioannidis, Paolo Ossola, Paolo Panaccio, Patricia Tejedor, Pedro Botelho, Pietro Fransvea, Pim B. Olthof, Pravin Meenashi Sundaram, Priscila Tanuri, Prokopis Christodoulou, Raffaele Galli, Raja Basit Khan, Rémy Kohler, Renan Colombari, Rogier Crolla, Sarah Vogler, Saulius Mikalauskas, Sameh Emile, Sergio Agradi, Sergio Larach, Sevket Baris Morkavuk, Simone Manfredelli, Spyridon Davakis, Stefano Gussago, Stefano Olmi, Stephan Bischofberger, Suman Baral, Syed Muhammad Ali, Tahar Fillali, Valentin Calu, Valentina Miacci, Venkatesh Munikrishnan, Vittoria Bellato, Vusal Aliyev, William Perry, Xenofon Papazarkadas, and Yasuko Maeda.
