# Supplementary material for: Surgeon preferences and practice patterns in rectopexy: Results of an international survey
Source: Colorectal Dis. 2026 Jan 4;28(1):e70355. doi: 10.1111/codi.70355 (PMC12765771; doi:10.1111/codi.70355)
Supplement: Supplementary file 1 — Appendix S1. [file CODI-28-0-s002.docx]

**Surgeon preferences and practice patterns in rectopexy: results of an international survey.**

**International Rectopexy Collaborative Group**

The following collaborators contributed to this study and are listed to ensure PubMed indexing:

Abdulahad Al-Ameri, Adam Mylonakis, Ahmed Abdelsamad, Aakansha Giri Goswami, Al Marazgh Mohammad, Alberto Aiolfi, Alessia Fassari, Angelo Alessandro Marra, Alessandro Garcea, Alexandra Menni, Alexandra Winter, Alexandros Chamzin, Alexandros Kozadinos, Alice Frontali, Amine Gouader, Amine Souadka, Amanda Pereira Lima, Andrea Balla, Andrea Peloso, Andrea Pierre Luzzi, Andreas Panagakis, Andrei Chitul, Andrei Popa, Angelo Stuto, Angeliki Vouchara, Antonio Castaldi, Antonio Luberto, Arcangelo Picciariello, Argyrios Ioannidis, Augustinas Bausys, Audrius Dulskas, Baljit Singh, Benjamin Fernandez, Boris Schiltz, Bruno Perotti, Bruno Roche, Brunella M. Pirozzi, Carlos Augusto Gomes, Carmen Gorgan, Céline Duvoisin Cordoba, Charles Sabbagh, Christina A. Fleming, Christos Barkolias, Christos Chouliaras, Christoph Werner Strey, Claudio Soravia, Cosimo R. Scarpa, Dan-Eduard Giuvara, Danilo Vinci, David Alessio Merlini, David D.E. Zimmerman, Debby Keller, Deiro Giacomo, Dieter Hahnloser, Diletta Corallino, Dimitris Korkolis, Dimitrios Linardoutsos, Dimitrios Moris, Dimitrios Schizas, Eleandros Kyros, Elisa Reitano, Elissavet Anestiadou, Emanuela Silva Alvarenga, Eric G. Weiss, Fabio Carbone, Federica Di Marco, Filippo Carannante, Flaviu Ionut Faur, Floryn Cherbanyk, Francesco Ferrara, Francesco Pata, Gaetano Florio, Gabriele Naldini, Gabriele Pozzo, Gabriella Teresa Capolupo, Giacomo Ambrogi, Giacomo Calini, Gianluca Pellino, Gianpiero Gravante, Giovanna Dasilva, Giovanni Cestaro, Giovanni Tebala, Giovanni Tomasicchio, Giuseppe Brisinda, Giuseppe Candilio, Giuseppe Giuliani, Goran Barisic, Goytom Knfe, George Stavrou, Georgios Korovesis, Georgios Peros, Georgios Tzikos, Gustavo Nari, Harish Neelamraju Lakshmi, Hemendra Kumar Mangal, Hugh M. Paterson, Ibrahim Burak Bahçecioğlu, Ibrahim Ethem Gecim, İlgar Ismayilov, Ioannis Katsaros, Jasper Stijns, Jelenko Jelenkovic, Jin Jiun Mah, Justin Davies, Kashish Malhotra, Klaus Peitgen, Konstantinos Tsimogiannis, Konstantinos Zarras, Lars Thomas Seeberg, Leandro Siragusa, Lorenzo Epis, Lucio Taglietti, Lysandros Karydakis, Maria Chiara Ranucci, Maria Sotiropoulou, Mark Potter, Marko Miladinov, Martin Bertrand, Matteo Santoliquido, Mhairi Collie, Michel Adamina, Michaela Ramser, Michaël Racine, Michail Vailas, Mohamed Ali Chaouch, Mohd. Azharuddin Attar, Mostafa Shalaby, Muhammad Rafaih Iqbal, Muhammad Umar Younis, Mustafa Yener Uzunoglu, Niels Komen, Nicola Colucci, Nicolas C. Buchs, Nir Horesh, Noam Shussman, Nora Abbesorabi, Nuri Okkabaz, Omer Yalkin, Orestis Ioannidis, Paolo Ossola, Paolo Panaccio, Patricia Tejedor, Pedro Botelho, Pietro Fransvea, Pim B. Olthof, Pravin Meenashi Sundaram, Priscila Tanuri, Prokopis Christodoulou, Raffaele Galli, Raja Basit Khan, Rémy Kohler, Renan Colombari, Rogier Crolla, Sarah Vogler, Saulius Mikalauskas, Sameh Emile, Sergio Agradi, Sergio Larach, Sevket Baris Morkavuk, Simone Manfredelli, Spyridon Davakis, Stefano Gussago, Stefano Olmi, Stephan Bischofberger, Suman Baral, Syed Muhammad Ali, Tahar Fillali, Valentin Calu, Valentina Miacci, Venkatesh Munikrishnan, Vittoria Bellato, Vusal Aliyev, William Perry, Xenofon Papazarkadas, and Yasuko Maeda.
