# Supplementary figures and images for: Surgeon preferences and practice patterns in rectopexy: Results of an international survey
Source: Colorectal Dis. 2026 Jan 4;28(1):e70355. doi: 10.1111/codi.70355 (PMC12765771; doi:10.1111/codi.70355)

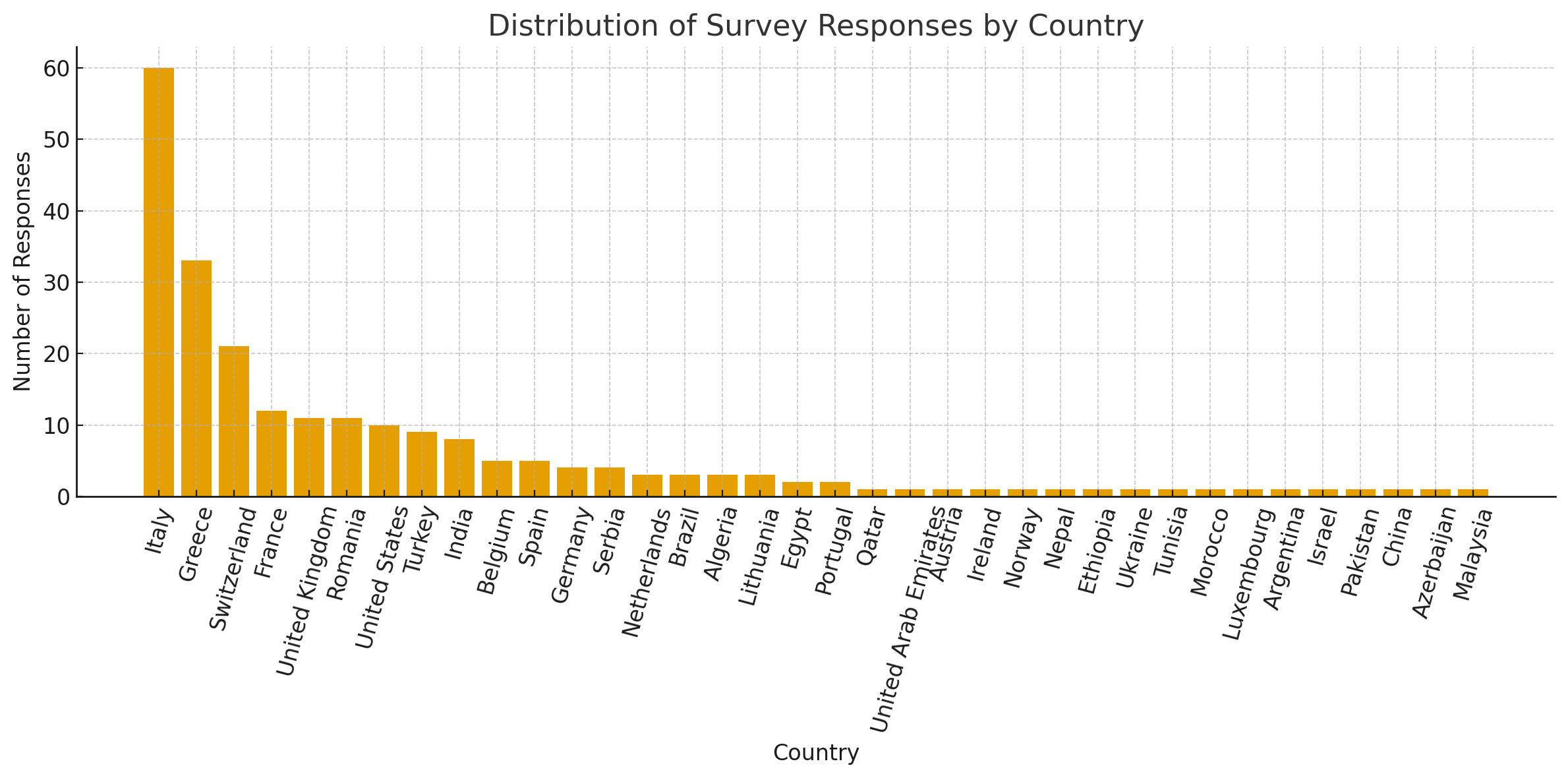

Supplement: Supplementary file 2 — Figure S1. [file CODI-28-0-s003.png]
