# Supplementary material for: Surgeon preferences and practice patterns in rectopexy: Results of an international survey
Source: Colorectal Dis. 2026 Jan 4;28(1):e70355. doi: 10.1111/codi.70355 (PMC12765771; doi:10.1111/codi.70355)
Supplement: Supplementary file 3 — Table S1. [file CODI-28-0-s001.docx]

**Supplementary Table 1:** **Pre-, intra-, and postoperative characteristics by world region**

| Variable | Category | | Europe n (%) | | Asia n (%) | | Africa n (%) | | North America n (%) | | South America n (%) | | p-value | |
| --- | --- | --- | --- | --- | --- | --- | --- | --- | --- | --- | --- | --- | --- | --- |
| Antibiotic Prophylaxis | IV | 147 (81.7%) | | 19 (79.2%) | | 5 (62.5%) | | 6 (60.0%) | | 3 (75.0%) | | 0.235 | |  |
|  | Oral | 19 (10.6%) | | 4 (16.7%) | | 2 (25.0%) | | 4 (40.0%) | | 1 (25.0%) | |  | |  |
|  | No | 14 (7.8%) | | 1 (4.2%) | | 1 (12.5%) | | 0 (0.0%) | | 0 (0.0%) | |  | |  |
| Mechanical Bowel Preparation | Enema | 70 (38.9%) | | 6 (25.0%) | | 4 (50.0%) | | 2 (20.0%) | | 1 (25.0%) | | 0.031 | |  |
|  | Oral | 70 (38.9%) | | 17 (70.8%) | | 4 (50.0%) | | 7 (70.0%) | | 1 (25.0%) | |  | |  |
|  | No | 40 (22.2%) | | 1 (4.2%) | | 0 (0.0%) | | 1 (10.0%) | | 2 (50.0%) | |  | |  |
| Preferred Surgical Approach | Open | 30 (16.7%) | | 5 (20.8%) | | 3 (37.5%) | | 2 (20.0%) | | 3 (75.0%) | | 0.001 | |  |
|  | Laparoscopic | 114 (63.3%) | | 18 (75.0%) | | 5 (62.5%) | | 2 (20.0%) | | 1 (25.0%) | |  | |  |
|  | Robotic | 36 (20.0%) | | 1 (4.2%) | | 0 (0.0%) | | 6 (60.0%) | | 0 (0.0%) | |  | |  |
| Type of Rectal Dissection | Anterior | 70 (38.9%) | | 9 (37.5%) | | 3 (37.5%) | | 4 (40.0%) | | 1 (25.0%) | | 0.987 | |  |
|  | Posterior | 110 (61.1%) | | 15 (62.5%) | | 5 (62.5%) | | 6 (60.0%) | | 3 (75.0%) | |  | |  |
|  | Lateral | 0 (0.0%) | | 0 (0.0%) | | 0 (0.0%) | | 0 (0.0%) | | 0 (0.0%) | |  | |  |
| Systematic Mesh Use | Yes | 122 (67.8%) | | 17 (70.8%) | | 6 (75.0%) | | 5 (50.0%) | | 4 (100.0%) | | 0.455 | |  |
|  | No | 58 (32.2%) | | 7 (29.2%) | | 2 (25.0%) | | 5 (50.0%) | | 0 (0.0%) | |  | |  |
| Mesh Type (if used) | Synthetic | 123 (68.3%) | | 15 (62.5%) | | 7 (87.5%) | | 4 (40.0%) | | 4 (100.0%) | | 0.154 | |  |
|  | Biologic | 28 (15.6%) | | 3 (12.5%) | | 0 (0.0%) | | 1 (10.0%) | | 0 (0.0%) | |  | |  |
|  | No | 29 (16.1%) | | 6 (25.0%) | | 1 (12.5%) | | 5 (50.0%) | | 0 (0.0%) | |  | |  |
| Distal Fixation (if mesh) | Absorbable | 79 (59.0%) | | 6 (50.0%) | | 2 (50.0%) | | 4 (80.0%) | | 0 (0.0%) | | 0.724 | |  |
|  | Non absorbable | 42 (31.3%) | | 5 (41.7%) | | 2 (50.0%) | | 1 (20.0%) | | 0 (0.0%) | |  | |  |
|  | Tacking | 11 (8.2%) | | 0 (0.0%) | | 0 (0.0%) | | 0 (0.0%) | | 0 (0.0%) | |  | |  |
|  | Glue | 2 (1.5%) | | 1 (8.3%) | | 0 (0.0%) | | 0 (0.0%) | | 0 (0.0%) | |  | |  |
| Promontory Fixation (if mesh) | Absorbable | 22 (14.7%) | | 2 (11.1%) | | 1 (14.3%) | | 0 (0.0%) | | 0 (0.0%) | | 0.608 | |  |
|  | Non absorbable | 74 (49.3%) | | 10 (55.6%) | | 2 (28.6%) | | 5 (83.3%) | | 4 (100.0%) | |  | |  |
|  | Tacking | 51 (34.0%) | | 5 (27.8%) | | 4 (57.1%) | | 1 (16.7%) | | 0 (0.0%) | |  | |  |
|  | Glue | 3 (2.0%) | | 1 (5.6%) | | 0 (0.0%) | | 0 (0.0%) | | 0 (0.0%) | |  | |  |
| Peritoneal Flap Closure | Yes | 162 (90.0%) | | 17 (70.8%) | | 6 (75.0%) | | 7 (70.0%) | | 4 (100.0%) | | 0.027 | |  |
|  | No | 18 (10.0%) | | 7 (29.2%) | | 2 (25.0%) | | 3 (30.0%) | | 0 (0.0%) | |  | |  |
| Drain Placement | Yes | 47 (26.1%) | | 15 (62.5%) | | 1 (12.5%) | | 3 (30.0%) | | 1 (25.0%) | | 0.005 | |  |
|  | No | 133 (73.9%) | | 9 (37.5%) | | 7 (87.5%) | | 7 (70.0%) | | 3 (75.0%) | |  | |  |
| Local Wound Infiltration | Yes | 104 (57.8%) | | 13 (54.2%) | | 1 (12.5%) | | 6 (60.0%) | | 4 (100.0%) | | 0.048 | |  |
|  | No | 76 (42.2%) | | 11 (45.8%) | | 7 (87.5%) | | 4 (40.0%) | | 0 (0.0%) | |  | |  |
| Urinary Catheter | Yes | 154 (85.6%) | | 19 (79.2%) | | 5 (62.5%) | | 10 (100.0%) | | 4 (100.0%) | | 0.177 | |  |
|  | No | 26 (14.4%) | | 5 (20.8%) | | 3 (37.5%) | | 0 (0.0%) | | 0 (0.0%) | |  | |  |
| Postoperative Laxatives | Yes | 82 (45.6%) | | 13 (54.2%) | | 2 (25.0%) | | 3 (30.0%) | | 1 (25.0%) | | 0.376 | |  |
|  | No | 47 (26.1%) | | 3 (12.5%) | | 1 (12.5%) | | 3 (30.0%) | | 2 (50.0%) | |  | |  |
|  | Selected | 51 (28.3%) | | 8 (33.3%) | | 5 (62.5%) | | 4 (40.0%) | | 1 (25.0%) | |  | |  |
| Postoperative Enema | Yes | 21 (11.7%) | | 3 (12.5%) | | 0 (0.0%) | | 0 (0.0%) | | 1 (25.0%) | | 0.534 | |  |
|  | No | 159 (88.3%) | | 21 (87.5%) | | 8 (100.0%) | | 10 (100.0%) | | 3 (75.0%) | |  | |  |
| Care Pathway | Hospitalisation | 157 (87.2%) | | 19 (79.2%) | | 8 (100.0%) | | 6 (60.0%) | | 2 (50.0%) | | 0.022 | |  |
|  | Ambulatory | 23 (12.8%) | | 5 (20.8%) | | 0 (0.0%) | | 4 (40.0%) | | 2 (50.0%) | |  | |  |
| Postoperative Physiotherapy | Yes | 65 (36.1%) | | 8 (33.3%) | | 5 (62.5%) | | 2 (20.0%) | | 3 (75.0%) | | 0.187 | |  |
|  | No | 115 (63.9%) | | 16 (66.7%) | | 3 (37.5%) | | 8 (80.0%) | | 1 (25.0%) | |  | |  |

**Supplementary Table 2:** **Pre-, intra-, and postoperative characteristics by surgical specialty**

| Variable | Category | General Surgeons  n (%) | Colorectal Surgeons  n (%) | p-value |
| --- | --- | --- | --- | --- |
| Antibiotic Prophylaxis | IV | 122 (81.3%) | 58 (76.3%) | 0.480 |
|  | Oral | 17 (11.3%) | 13 (17.1%) |  |
|  | No | 11 (7.3%) | 5 (6.6%) |  |
| Mechanical Bowel Preparation | Enema | 55 (36.7%) | 28 (36.8%) | 0.134 |
|  | Oral | 71 (47.3%) | 28 (36.8%) |  |
|  | No | 24 (16.0%) | 20 (26.3%) |  |
| Preferred Surgical Approach | Open | 39 (26.0%) | 4 (5.3%) | 0.000 |
|  | Laparoscopic | 97 (64.7%) | 43 (56.6%) |  |
|  | Robotic | 14 (9.3%) | 29 (38.2%) |  |
| Type of Rectal Dissection | Anterior | 51 (34.0%) | 36 (47.4%) | 0.060 |
|  | Posterior | 99 (66.0%) | 40 (52.6%) |  |
|  | Lateral | 0 (0.0%) | 0 (0.0%) |  |
| Systematic Mesh Use | Yes | 96 (64.0%) | 58 (76.3%) | 0.070 |
|  | No | 54 (36.0%) | 18 (23.7%) |  |
| Mesh Type (if used) | Synthetic | 101 (67.3%) | 52 (68.4%) | 0.458 |
|  | Biologic | 19 (12.7%) | 13 (17.1%) |  |
|  | No | 30 (20.0%) | 11 (14.5%) |  |
| Distal Fixation (if mesh) | Absorbable | 54 (57.4%) | 37 (60.7%) | 0.851 |
|  | Non absorbable | 30 (31.9%) | 20 (32.8%) |  |
|  | Tacking | 8 (8.5%) | 3 (4.9%) |  |
|  | Glue | 2 (2.1%) | 1 (1.6%) |  |
| Promontory Fixation (if mesh) | Absorbable | 21 (17.9%) | 4 (5.9%) | 0.094 |
|  | Non absorbable | 59 (50.4%) | 36 (52.9%) |  |
|  | Tacking | 34 (29.1%) | 27 (39.7%) |  |
|  | Glue | 3 (2.6%) | 1 (1.5%) |  |
| Peritoneal Flap Closure | Yes | 127 (84.7%) | 69 (90.8%) | 0.221 |
|  | No | 23 (15.3%) | 7 (9.2%) |  |
| Drain Placement | Yes | 55 (36.7%) | 12 (15.8%) | 0.001 |
|  | No | 95 (63.3%) | 64 (84.2%) |  |
| Local Wound Infiltration | Yes | 76 (50.7%) | 52 (68.4%) | 0.015 |
|  | No | 74 (49.3%) | 24 (31.6%) |  |
| Urinary Catheter | Yes | 128 (85.3%) | 64 (84.2%) | 0.845 |
|  | No | 22 (14.7%) | 12 (15.8%) |  |
| Postoperative Laxatives | Yes | 60 (40.0%) | 41 (53.9%) | 0.082 |
|  | No | 43 (28.7%) | 13 (17.1%) |  |
|  | Selected | 47 (31.3%) | 22 (28.9%) |  |
| Postoperative Enema | Yes | 19 (12.7%) | 6 (7.9%) | 0.371 |
|  | No | 131 (87.3%) | 70 (92.1%) |  |
| Care Pathway | Hospitalisation | 124 (82.7%) | 68 (89.5%) | 0.237 |
|  | Ambulatory | 26 (17.3%) | 8 (10.5%) |  |
| Postoperative Physiotherapy | Yes | 59 (39.3%) | 24 (31.6%) | 0.307 |
|  | No | 91 (60.7%) | 52 (68.4%) |  |

**Supplementary Table 3: Pre-, intra-, and postoperative characteristics by years of specialised practice**

| Variable | Category | <10 years n (%) | 10-20 years n (%) | 20-30 years n (%) | p-value |
| --- | --- | --- | --- | --- | --- |
| Antibiotic Prophylaxis | IV | 124 (83.8%) | 33 (73.3%) | 23 (69.7%) | 0.007 |
|  | Oral | 13 (8.8%) | 7 (15.6%) | 10 (30.3%) |  |
|  | No | 11 (7.4%) | 5 (11.1%) | 0 (0.0%) |  |
| Mechanical Bowel Preparation | Enema | 59 (39.9%) | 15 (33.3%) | 9 (27.3%) | 0.413 |
|  | Oral | 64 (43.2%) | 21 (46.7%) | 14 (42.4%) |  |
|  | No | 25 (16.9%) | 9 (20.0%) | 10 (30.3%) |  |
| Preferred Surgical Approach | Open | 27 (18.2%) | 7 (15.6%) | 9 (27.3%) | 0.664 |
|  | Laparoscopic | 93 (62.8%) | 30 (66.7%) | 17 (51.5%) |  |
|  | Robotic | 28 (18.9%) | 8 (17.8%) | 7 (21.2%) |  |
| Type of Rectal Dissection | Anterior | 52 (35.1%) | 22 (48.9%) | 13 (39.4%) | 0.250 |
|  | Posterior | 96 (64.9%) | 23 (51.1%) | 20 (60.6%) |  |
|  | Lateral | 0 (0.0%) | 0 (0.0%) | 0 (0.0%) |  |
| Systematic Mesh Use | Yes | 99 (66.9%) | 33 (73.3%) | 22 (66.7%) | 0.705 |
|  | No | 49 (33.1%) | 12 (26.7%) | 11 (33.3%) |  |
| Mesh Type (if used) | Synthetic | 102 (68.9%) | 29 (64.4%) | 22 (66.7%) | 0.379 |
|  | Biologic | 19 (12.8%) | 10 (22.2%) | 3 (9.1%) |  |
|  | No | 27 (18.2%) | 6 (13.3%) | 8 (24.2%) |  |
| Distal Fixation (if mesh) | Absorbable | 59 (59.0%) | 19 (54.3%) | 13 (65.0%) | 0.504 |
|  | Non absorbable | 29 (29.0%) | 14 (40.0%) | 7 (35.0%) |  |
|  | Tacking | 10 (10.0%) | 1 (2.9%) | 0 (0.0%) |  |
|  | Glue | 2 (2.0%) | 1 (2.9%) | 0 (0.0%) |  |
| Promontory Fixation (if mesh) | Absorbable | 20 (16.7%) | 4 (10.0%) | 1 (4.0%) | 0.001 |
|  | Non absorbable | 72 (60.0%) | 14 (35.0%) | 9 (36.0%) |  |
|  | Tacking | 27 (22.5%) | 20 (50.0%) | 14 (56.0%) |  |
|  | Glue | 1 (0.8%) | 2 (5.0%) | 1 (4.0%) |  |
| Peritoneal Flap Closure | Yes | 132 (89.2%) | 38 (84.4%) | 26 (78.8%) | 0.248 |
|  | No | 16 (10.8%) | 7 (15.6%) | 7 (21.2%) |  |
| Drain Placement | Yes | 44 (29.7%) | 13 (28.9%) | 10 (30.3%) | 0.990 |
|  | No | 104 (70.3%) | 32 (71.1%) | 23 (69.7%) |  |
| Local Wound Infiltration | Yes | 90 (60.8%) | 22 (48.9%) | 16 (48.5%) | 0.218 |
|  | No | 58 (39.2%) | 23 (51.1%) | 17 (51.5%) |  |
| Urinary Catheter | Yes | 127 (85.8%) | 37 (82.2%) | 28 (84.8%) | 0.840 |
|  | No | 21 (14.2%) | 8 (17.8%) | 5 (15.2%) |  |
| Postoperative Laxatives | Yes | 61 (41.2%) | 28 (62.2%) | 12 (36.4%) | 0.043 |
|  | No | 39 (26.4%) | 5 (11.1%) | 12 (36.4%) |  |
|  | Selected | 48 (32.4%) | 12 (26.7%) | 9 (27.3%) |  |
| Postoperative Enema | Yes | 19 (12.8%) | 4 (8.9%) | 2 (6.1%) | 0.465 |
|  | No | 129 (87.2%) | 41 (91.1%) | 31 (93.9%) |  |
| Care Pathway | Hospitalisation | 120 (81.1%) | 41 (91.1%) | 31 (93.9%) | 0.076 |
|  | Ambulatory | 28 (18.9%) | 4 (8.9%) | 2 (6.1%) |  |
| Postoperative Physiotherapy | Yes | 59 (39.9%) | 13 (28.9%) | 11 (33.3%) | 0.372 |
|  | No | 89 (60.1%) | 32 (71.1%) | 22 (66.7%) |  |

**Supplementary Table 4: Pre-, intra-, and postoperative characteristics by case volume**

| Variable | Category | <50 cases n (%) | 50-100 cases n (%) | 100-200 cases n (%) | >200 cases n (%) | p-value |
| --- | --- | --- | --- | --- | --- | --- |
| Antibiotic Prophylaxis | IV | 132 (81.0%) | 26 (78.8%) | 12 (66.7%) | 10 (83.3%) | 0.761 |
|  | Oral | 19 (11.7%) | 5 (15.2%) | 4 (22.2%) | 2 (16.7%) |  |
|  | No | 12 (7.4%) | 2 (6.1%) | 2 (11.1%) | 0 (0.0%) |  |
| Mechanical Bowel Preparation | Enema | 56 (34.4%) | 16 (48.5%) | 7 (38.9%) | 4 (33.3%) | 0.189 |
|  | Oral | 78 (47.9%) | 12 (36.4%) | 4 (22.2%) | 5 (41.7%) |  |
|  | No | 29 (17.8%) | 5 (15.2%) | 7 (38.9%) | 3 (25.0%) |  |
| Preferred Surgical Approach | Open | 32 (19.6%) | 4 (12.1%) | 5 (27.8%) | 2 (16.7%) | 0.000 |
|  | Laparoscopic | 112 (68.7%) | 18 (54.5%) | 6 (33.3%) | 4 (33.3%) |  |
|  | Robotic | 19 (11.7%) | 11 (33.3%) | 7 (38.9%) | 6 (50.0%) |  |
| Type of Rectal Dissection | Anterior | 55 (33.7%) | 16 (48.5%) | 10 (55.6%) | 6 (50.0%) | 0.120 |
|  | Posterior | 108 (66.3%) | 17 (51.5%) | 8 (44.4%) | 6 (50.0%) |  |
|  | Lateral | 0 (0.0%) | 0 (0.0%) | 0 (0.0%) | 0 (0.0%) |  |
| Systematic Mesh Use | Yes | 103 (63.2%) | 28 (84.8%) | 13 (72.2%) | 10 (83.3%) | 0.058 |
|  | No | 60 (36.8%) | 5 (15.2%) | 5 (27.8%) | 2 (16.7%) |  |
| Mesh Type (if used) | Synthetic | 108 (66.3%) | 24 (72.7%) | 13 (72.2%) | 8 (66.7%) | 0.580 |
|  | Biologic | 21 (12.9%) | 6 (18.2%) | 2 (11.1%) | 3 (25.0%) |  |
|  | No | 34 (20.9%) | 3 (9.1%) | 3 (16.7%) | 1 (8.3%) |  |
| Distal Fixation (if mesh) | Absorbable | 60 (57.1%) | 16 (64.0%) | 8 (57.1%) | 7 (63.6%) | 0.910 |
|  | Non absorbable | 35 (33.3%) | 7 (28.0%) | 4 (28.6%) | 4 (36.4%) |  |
|  | Tacking | 8 (7.6%) | 2 (8.0%) | 1 (7.1%) | 0 (0.0%) |  |
|  | Glue | 2 (1.9%) | 0 (0.0%) | 1 (7.1%) | 0 (0.0%) |  |
| Promontory Fixation (if mesh) | Absorbable | 22 (16.8%) | 2 (6.7%) | 1 (7.1%) | 0 (0.0%) | 0.186 |
|  | Non absorbable | 70 (53.4%) | 16 (53.3%) | 6 (42.9%) | 3 (30.0%) |  |
|  | Tacking | 36 (27.5%) | 11 (36.7%) | 7 (50.0%) | 7 (70.0%) |  |
|  | Glue | 3 (2.3%) | 1 (3.3%) | 0 (0.0%) | 0 (0.0%) |  |
| Peritoneal Flap Closure | Yes | 142 (87.1%) | 28 (84.8%) | 15 (83.3%) | 11 (91.7%) | 0.906 |
|  | No | 21 (12.9%) | 5 (15.2%) | 3 (16.7%) | 1 (8.3%) |  |
| Drain Placement | Yes | 57 (35.0%) | 6 (18.2%) | 2 (11.1%) | 2 (16.7%) | 0.042 |
|  | No | 106 (65.0%) | 27 (81.8%) | 16 (88.9%) | 10 (83.3%) |  |
| Local Wound Infiltration | Yes | 91 (55.8%) | 21 (63.6%) | 10 (55.6%) | 6 (50.0%) | 0.819 |
|  | No | 72 (44.2%) | 12 (36.4%) | 8 (44.4%) | 6 (50.0%) |  |
| Urinary Catheter | Yes | 136 (83.4%) | 30 (90.9%) | 16 (88.9%) | 10 (83.3%) | 0.693 |
|  | No | 27 (16.6%) | 3 (9.1%) | 2 (11.1%) | 2 (16.7%) |  |
| Postoperative Laxatives | Yes | 71 (43.6%) | 20 (60.6%) | 6 (33.3%) | 4 (33.3%) | 0.058 |
|  | No | 47 (28.8%) | 2 (6.1%) | 3 (16.7%) | 4 (33.3%) |  |
|  | Selected | 45 (27.6%) | 11 (33.3%) | 9 (50.0%) | 4 (33.3%) |  |
| Postoperative Enema | Yes | 22 (13.5%) | 2 (6.1%) | 0 (0.0%) | 1 (8.3%) | 0.246 |
|  | No | 141 (86.5%) | 31 (93.9%) | 18 (100.0%) | 11 (91.7%) |  |
| Care Pathway | Hospitalisation | 135 (82.8%) | 29 (87.9%) | 17 (94.4%) | 11 (91.7%) | 0.477 |
|  | Ambulatory | 28 (17.2%) | 4 (12.1%) | 1 (5.6%) | 1 (8.3%) |  |
| Postoperative Physiotherapy | Yes | 64 (39.3%) | 10 (30.3%) | 7 (38.9%) | 2 (16.7%) | 0.369 |
|  | No | 99 (60.7%) | 23 (69.7%) | 11 (61.1%) | 10 (83.3%) |  |
